# Auditory performance and language skills in children with auditory brainstem implants and cochlear implants

**DOI:** 10.1007/s00405-024-08594-0

**Published:** 2024-04-04

**Authors:** Nuriye Yıldırım Gökay, Beyza Demirtaş, Merve Özbal Batuk, Esra Yücel, Gonca Sennaroğlu

**Affiliations:** 1 Department of Audiology, Faculty of Healthy Science, Gazi University, Emek, 06900 Ankara, Turkey; 2 Department of Audiology, Faculty of Health Sciences, Hacettepe University, Ankara, Turkey

**Keywords:** Auditory brainstem implants, Cochlear implants, Children, Auditory performance, Language

## Abstract

**Purpose:**

This study aims to evaluate school-age language skills and auditory performance in different listening situations in children with cochlear implants and auditory brainstem implants.

**Method:**

The study included 60 children between the ages of 5 and 9 years with cochlear implants (CI) and auditory brainstem implants (ABI). The volunteer children were divided into two groups: bimodal CI-ABI and bilateral CI users. Test of Language Development: Primary (TOLD-P:4), which assesses components of language such as phonology, morphology, syntax and semantics, was used to evaluate school-age language skills. Children’s Auditory Performance Scale (CHAPS) was used to measure their listening performance in quiet, noisy, multi-stimulus environments and their auditory attention and memory skills in daily life. The correlations between language and auditory performance were analyzed and compared between the two groups.

**Results:**

Children with ABI showed poorer performance in school-age language skills and auditory performance in different listening environments (*p* < 0.05). Significant correlations were between school-age language skills and auditory performance (*p* < 0.05).

**Conclusion:**

Improved auditory performance is crucial for the development of school-age language skills. To improve auditory performance in children with ABI in different listening environments, assistive listening devices, acoustic environmental arrangements, informative activities, etc., should be used.

## Introduction

A pre-lingual severe and profound hearing loss has a negative impact on language and learning development [5]. Cochlear implants (CI) improve auditory performance and language skills in children with severe to profound sensorineural hearing loss [22]. However, CI is limited in auditory rehabilitation in cases of anatomical malformations in the inner ear and/or auditory nerve. Auditory brainstem implantation (ABI) is the preferred option in cases in which cochlear implantation is contraindicated due to these malformations [16, 17, 19]. Auditory brainstem implants help provide a sense of hearing by placing them in the cochlear nuclei in the brainstem without connecting to the inner ear [21].

Studies have demonstrated that auditory perception and language skills improve in children with CI and ABI [2, 18, 20]. A study revealed that the word recognition scores were 80% in a quiet environment and 45% in a noisy environment approximately 10 years after implantation. Similarly, 60% of children with cochlear implants can make a phone call to a familiar speaker but continue to have problems with complex language structures such as syntax, semantics, and pragmatics. A systematic review of the change of speech perception with cochlear implantation showed that children experienced a sharp improvement in early speech perception in 1 year after implantation. Children who are younger than 18 months benefit from CI in terms of speech recognition faster [3, 10, 14, 27]. On the other hand, ABI develops spoken language in children, but this happens slowly and gradually. A study comparing language characteristics between ABI and CI users showed that language development the ability to recognize two-syllable words and sentences are worse in children with ABI.

Language and communication skills in children with ABI develop in postoperative 12 months [15]. So, it has been shown that hearing loss can affect the quality of life by affecting speech and language development. The children with hearing loss have lower scores on quality of life than other children. Also, children with hearing loss have a higher risk of impaired language development and social life. Although it is promising that children with HL show similar levels of self-esteem and mental health as children with normal hearing, HL can negatively affect the quality of life of these children in various aspects (for example, personal relationships with other people and environmental/situational factors that challenge them). Noisy environments, distorted and/or distant auditory signals and hearing loss require children to use explicit processing mechanisms and high cognitive resources.

There are a limited number of studies that include functional assessment of hearing quality and daily life hearing performance in children using CI and ABI [1, 7, 10, 14]. According to these studies, although patients are unhappy with their ABI in some communication skills, overall, their quality of life improves over time. A study revealed that ABIs are suitable for children with cochlear anomalies to provide auditory input and benefit all developmental areas [2]. It has been found that children using ABI perform poorly compared to their peers using CI in terms of cognitive and language skills and daily life hearing performance [27]. To the authors’ best knowledge, there are no studies investigating language skills with the “Children’s Auditory Performance Scale (CHAPS)” in children with ABI in the same study. The CHAPS is generally addressed in the evaluation of central auditory processing disorder and validity–reliability studies [3, 4, 9, 23].

The current study aims to evaluate language skills and auditory performance in quiet-noisy environments in daily life and listening situations requiring auditory attention and auditory memory for children with CI and ABI. For this purpose, it is assumed that it will shed light on these berms of examining daily life listening performance and language skills in children with ABI, especially in a significant sample size.

## Materials and methods

This study was approved by The University Clinical Research Ethics Committee with GO23/601 decision number. All informed consent forms were obtained from all children and their parents.

### Participants

Children included in this study consisted of patients who applied to the University Department of Audiology. The volunteer children aged 5 to 9 years were divided into two groups: those using bilateral CI (*n* = 30) and those using bimodal CI-ABI (*n* = 30). An experienced radiologist and otologist diagnosed inner ear malformations using high-resolution computed tomography using axial sections. Auditory brainstem implantation is applied on children who have contraindications to cochlear implants due to inner ear and/or auditory nerve malformations. The children whose hearing loss diagnosis age and hearing aid starting age were less than 1 year, who had their first auditory implantation surgery before the age of 2, and who had regular use for at least 1 year after the activation of the auditory implant were included. Bilateral cochlear implanted children underwent simultaneous bilateral implantation surgery. For bimodal CI-ABI users, the time between two surgeries is, at most 2 years. Children with bilateral CI have no inner ear and/or auditory nerve anomalies. All participants receive regular auditory rehabilitation. In the evaluation of candidates for auditory implantation, additional disabilities in the fields of child psychiatry, developmental pediatrics, neurology, etc., are routinely examined before surgery by experts in the field. As a result, children diagnosed with additional deficiency in these areas or syndromic hearing losses were excluded from the study. The average free-field hearing thresholds of all children with bilateral auditory implants are approximately 25 to 45 dB HL at 500, 1000, and 2000 Hz. None of the children use FM systems or other assistive listening devices.

### Children’s Auditory Performance Scale (CHAPS)

The CHAPS is a 36-item questionnaire that compares a child’s listening behavior with other children of similar age and background in six different domains: listening in noisy, quiet, ideal, multiple-input conditions, and listening activities that require auditory memory/sequencing and auditory attention span. Each item is scored using a seven-point scale from + 1 to 5 (+ 1 = less difficulty than other children, 0 = same amount of problem, 1 = slightly more difficulty, 2 = more difficulty, 3 = important significantly more difficulty, 4 = significantly more difficulty, and 5 = unable to function at all). There are seven items each assessing listening in noise and quiet, three assessing listening in ideal and multiple-input conditions, and eight items each for listening that requires auditory memory/sequence and attention span. The items in the subsections include questions such as “when asked a question, when given simple commands, when given more than one command, when with several children, when listening in a room with visual stimuli, etc.”. As mentioned above, these are asked to be scored according to the degree of difficulty of auditory performance. The “average part score” for each part was calculated by dividing the total score of the items in each part by the number of items in the part. The “average total score” was calculated by dividing the total score by 36. In this study, not the average scores, but the total score of each section and the scale’s total score were analyzed statistically. The studies with the CHAPS in different languages are examined, it is emphasized that the current scale is a safe and appropriate tool for measuring hearing performance [3, 4, 9, 23]. Based on that there are no studies evaluating children with ABI and CI. According to the authors’ best knowledge, it aims to make a unique contribution to the literature.

### Test of Language Development: Primary (TOLD-P:4)

The TOLD-P:4 test was used for the general assessment of children’s school-age language skills. This test is a standard test whose validity and reliability have been studied and is used in many studies and clinical applications [12, 24, 26, 28]. This test includes six basic skills: showing the picture of the spoken word, explaining the relationship between two words, describing a word, showing the picture of the spoken sentence, repeating the spoken sentence, and completing the morphemes in a sentence. The sum of the scores of these tests reveals the verbal language score. In this test, 1 point is given for each correct answer.

### Statistical analysis

All statistical analyzes were implemented by SPSS Statistics v.23.0. The normal distribution of the data was examined using histogram graphs and analytical methods. The descriptive statistics were presented as mean and standard deviation for normally distributed data, and as median and range for non-normally distributed data. The comparisons between groups with bilateral CI and bimodal CI-ABI were evaluated by independent samples *t* test or Mann–Whitney *U* test. The relationship between TOLD-P:4 and CHAPS scores was examined by correlation analysis. Statistical significance was set at *p* < 0.05.

## Results

A total of 60 volunteer children and their families, 30 (16 girls, 14 boys) with CI and 30 (15 boys, 15 girls) with ABI, were included in the study. The mean age was 90.40 ± 9.01 months in the CI group and 91.87 ± 7.77 months in the ABI group. The age at onset of hearing loss was 5.07 ± 1.34 months in the CI group and 4.43 ± 1.25 months in the ABI group. The duration of cochlear implant use was 19.00 ± 3.41 months in the CI group and 20.23 ± 3.87 months in the ABI group. The etiologies of hearing loss were generally idiopathic. The educational level of the children’s families was predominantly high school and university. There were no statistically significant differences between the ABI and CI groups in terms of age of hearing loss, age of first implantation, age of starting hearing aid use, and duration of implant use. See Tables 1 and 2 for detailed information on demographic information.

**Table 1:** Demographic information I

	Groups				*p*
	ABI		CI		
	Mean	Standard deviation	Mean	Standard deviation	
Age (months)	91.87	7.77	90.40	9.01	0.502
Onset age of hearing loss (months)	4.43	1.25	5.07	1.34	0.063
Onset age of hearing aid usage (months)	5.50	0.86	5.40	1.13	0.196
Age of cochlear implantation (months)	20.23	3.87	19.00	3.41	0.253
Duration of cochlear implant (months)	71.03	7.40	71.80	9.89	0.702

**Table 2:** Demographic information II

	Groups	
	ABI	CI
	Count	Count
Gender		
Girl	15	16
Boy	15	14
Etiology of hearing loss		
İdiopathic	16	16
Other reason	14	14
Modality of cochlear implantation		
Bilateral simultaneously cochlear implant	0	30
Bimodal cochlear implant and auditory brainstem implant	30	0
Family education level		
Primary school	0	0
High school	14	20
University	16	10

The CHAPS total scores were 56.30 ± 12.80 in the ABI group and 35.80 ± 11.58 in the CI group. According to listening performance in noisy environments, children with ABI had 11.43 ± 4.46 points and children with CI had 8.66 ± 3.39 points. The statistically significant differences were found between children with ABI and CI in terms of CHAPS total score and auditory attention and auditory memory skills in quiet and noisy listening conditions (see Table 3). According to the instructions of the CHAPS, the higher the score, the more difficult the child had. The children with ABI showed poorer performance in listening situations in daily life and skills requiring auditory memory-attention.

**Table 3:** The scores of total CHAPS and sections

	Groups	*N*	Mean	Std. deviation	*p*
CHAPS total	ABI	30	56.30	12.80	< 0.001*
	CI	30	35.80	11.58	
CHAPS attention	ABI	30	16.10	6.58	< 0.001*
	CI	30	9.76	3.21	
CHAPS memory	ABI	30	19.06	7.02	< 0.001*
	CI	30	12.63	5.51	
CHAPS noisy environments	ABI	30	11.43	4.46	0.009*
	CI	30	8.66	3.39	
CHAPS silent environments	ABI	30	5.90	2.83	0.003*
	CI	30	3.93	1.92	

*There is a statistically significant difference

The statistically significant differences were found between the groups in all TOLD-P:4 subtests and verbal language scores in school-age language skills in children with ABI and CI (*p* < 0.001). Accordingly, while the TOLD-P:4 verbal language score of children with ABI is 67.30 ± 6.13, it is 77.30 ± 5.90 in children with CI.

The correlation analyses were conducted between children’s school-age language scores and the CHAPS scores. Accordingly, there are strong, statistically significant negative correlations between the CHAPS auditory attention and auditory memory scores and TOLD-P:4 verbal language scores (see Table 4). According to the CHAPS scoring guideline, there is a negative relationship between the TOLD-P:4 score because a higher CHAPS score indicates poorer performance.

**Table 4:** Correlations I

	TOLD:P-4 verbal language	CHAPS attention	CHAPS memory
TOLD:P-4 verbal language			
*r*	1	− 0.695**	− 0.640**
*p*		< 0.001	< 0.001
*N*	40	40	40
CHAPS attention			
*r*	− 0.695**	1	0.601**
*p*	< 0.001		< 0.001
*N*	40	60	60
CHAPS memory			
*r*	− 0.640**	0.601**	1
*p*	< 0.001	< 0.001	
*N*	40	60	60

**Correlation is significant at the 0.01 level (2-tailed). *r* Pearson correlation

A very strong negative, statistically significant correlation was detected between the CHAPS total score and the TOLD-P:4 verbal language score (*r* = − 0.851, *p* < 0.001). Similarly, moderate statistically significant correlations were found between auditory performance in the CHAPS quiet and noisy listening conditions and TOLD-P:4 verbal language score (see Table 5).

**Table 5:** Correlations II

	TOLD:P-4 verbal language	CHAPS total	CHAPS noisy environments	CHAPS silent environments
TOLD:P-4 verbal language				
*r*	1	− 0.851**	− 0.508**	− 0.534**
*p*		< 0.001	0.001	< 0.001
*N*	40	40	40	40
CHAPS total				
*r*	− 0.851**	1	0.603**	0.579**
*p*	< 0.001		< 0.001	< 0.001
*N*	40	60	60	60
CHAPS noisy environments				
*r*	− 0.508**	0.603**	1	0.773**
*p*	0.001	< 0.001		< 0.001
*N*	40	60	60	60
CHAPS silent environments				
*r*	− 0.534**	0.579**	0.773**	1
*p*	< 0.001	< 0.001	< 0.001	
*N*	40	60	60	60

**Correlation is significant at the 0.01 level (2-tailed). *r* Pearson correlation

**Fig. 1:**
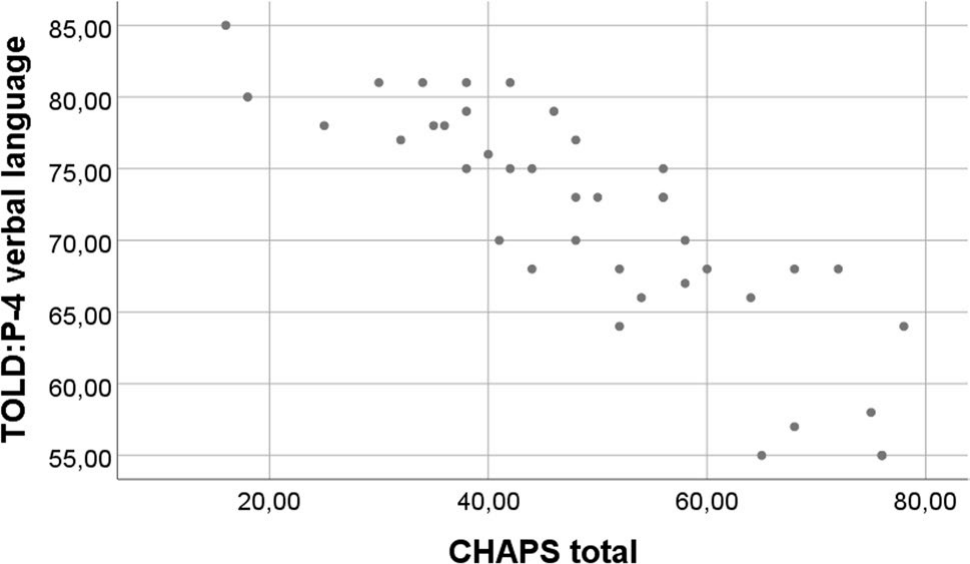
The scatter graph of correlation

Additionally, Fig. 1 shows the negative and strong relationship between the TOLD-P:4 verbal language score and the CHAPS total score.

## Discussion

This study investigated auditory performance and language skills in routine listening conditions, such as quiet, ideal, noisy, long-distance, etc., in children with bimodal (CI-ABI) and bilateral cochlear implants. It has been determined that there is a significant relationship between school-age language skills and hearing quality in children with auditory implants. The daily life auditory performance and school-age language skills were found to be poorer in children with auditory brainstem implants.

There are active and changing listening environments in daily life. Children with auditory implants struggle to maintain joint attention, use selective attention skills toward the target sound source, and focus on changing conditions. This can be predicted from the scores given to the questions about listening environments in the subsections of the CHAPS scale used in the current study. Thanks to the CHAPS subsections detailed in the method section, attention and memory performances underlying routine skills such as maintaining a conversation, executing multiple commands, and answering questions can be assessed. The present study assumes a unique contribution to the literature in terms of investigating auditory performance in quiet, noisy, ideal, multi-stimulus environments and environments requiring auditory attention-memory [1, 7, 10, 14]. Similar to other studies assessing hearing quality of life and daily life hearing performance, this study also showed that children with the ABI were poorer [1, 14, 27]. Possible reasons for this finding may be the lack of auditory stimuli in the preoperative period in children with ABI, different tonotopic organization of the ABI placement site, inability to reach optimum hearing thresholds due to postoperative fitting and follow-up difficulties, and differences in the experience of specialists [2, 17, 21, 30, 31]. On the other hand, in children with indications for ABI, such as auditory nerve and inner ear anomalies, auditory rehabilitation with ABI has been found to significantly improve children’s quality of life and daily life hearing performance [1, 7].

The children who cannot fully receive the auditory stimulus may appear in various ways, such as being more easily distracted, out of context, unable to focus on the target speaker, etc. [6, 13]. This results in more difficulties in developing and using verbal language. In the present study, children who had limited exposure to auditory stimuli during the critical period of 0–2 years of life [29], despite being implanted at a relatively early age, may have performed poorly on the TOLD-P:4 school-age language test for this reason. On the other hand, the lower language scores of children using ABI may be due to the preoperative and postoperative follow-up and rehabilitation difficulties of the ABI process, less audibility in the preoperative period, and the failure of postoperative fitting sessions to achieve good hearing thresholds [18, 21, 27, 31].

The correlations between school-age language skills and auditory performances in various listening environments are consistent with the study hypotheses. Thus, children with limited auditory access, who do not receive sufficient auditory input in routine listening environments, may develop limited verbal language. Also, there are several studies evaluating hearing performance and language skills in ABI users [1, 7, 14, 27]. The present findings are consistent with these studies. Although children with ABI show limited development compared to their peers with CI, they offer significant progress in language and communication skills thanks to ABI [8, 11, 25].

To the best of the authors’ knowledge, the CHAPS scale, which assesses skills requiring auditory attention and auditory memory in silence, noise, and multi-stimulus environments, has not been applied in children with ABI. In addition, another unique aspect of the study is the comparison of the auditory performance in different conditions with school-age language skills. The study’s strengths include the homogeneity of the children in terms of age at implantation, age at diagnosis of hearing loss, age at onset of hearing aid use, etc., and the inclusion of a relatively large sample. On the other hand, future studies with methods that include cognitive tests and high-level auditory processing tests are needed.

## Conclusion

In the present study, children with bimodal CI-ABI performed poorly in terms of language skills and auditory skills in different listening environments compared to their peers with bilateral CI. Access to auditory stimuli and improved auditory performance are crucial for improved school-age language skills. It may be helpful to recommend using assistive listening devices to improve auditory performance in children with ABI. Similarly, acoustic modifications at school, home, and other listening environments can improve auditory performance. Additionally, informative activities about children with ABI and their auditory performance should be organized for families and teachers. Moreover, it is recommended to be more attentive in the diagnosis and follow-up of children with ABIs, to consult experienced specialists, and to work as a multidisciplinary team.

## Data Availability

The current study’s data are kept secure and confidential with the first author. The data can be shared when necessary.
